# Targeted disruption of the aralkylamine *N*-acetyltransferase gene in a seasonal mammal, *Mesocricetus auratus*

**DOI:** 10.1093/pnasnexus/pgaf159

**Published:** 2025-05-20

**Authors:** Junko Kawabe, Natsumi Kawakami, Michiko Hirose, Yukari Kitamura, Mamoru Nagano, Yusuke Maruyama, Yohei Matsuyama, Teruki Hamano, Satoshi Koinuma, Takahiko Shiina, Momoko Kobayashi, Ritsuko Matsumura, Atsuhiko Hattori, Yasufumi Shigeyoshi, Atsuo Ogura, Koichi Node, Makoto Akashi

**Affiliations:** The Research Institute for Time Studies, Yamaguchi University, 1677-1 Yoshida, Yamaguchi, Yamaguchi 753-8511, Japan; The Research Institute for Time Studies, Yamaguchi University, 1677-1 Yoshida, Yamaguchi, Yamaguchi 753-8511, Japan; RIKEN BioResource Research Center, 3-1-1 Koyadai, Tsukuba, Ibaraki 305-0074, Japan; The Research Institute for Time Studies, Yamaguchi University, 1677-1 Yoshida, Yamaguchi, Yamaguchi 753-8511, Japan; Department of Anatomy and Neurobiology, Kindai University, 377-2 Ohno-Higashi, Osaka-Sayama, Osaka 589-8511, Japan; Department of Biology, College of Liberal Arts and Sciences, Tokyo Medical and Dental University, Ichikawa, Chiba 272-0827, Japan; Department of Sport and Wellness, College of Sport and Wellness, Rikkyo University, Niiza, Saitama 352-8558, Japan; The Research Institute for Time Studies, Yamaguchi University, 1677-1 Yoshida, Yamaguchi, Yamaguchi 753-8511, Japan; The Research Institute for Time Studies, Yamaguchi University, 1677-1 Yoshida, Yamaguchi, Yamaguchi 753-8511, Japan; Department of Anatomy and Neurobiology, Kindai University, 377-2 Ohno-Higashi, Osaka-Sayama, Osaka 589-8511, Japan; Department of Basic Veterinary Science, Laboratory of Physiology, Joint Department of Veterinary Medicine, Faculty of Applied Biological Sciences, Gifu University, 1-1 Yanagido, Gifu, Gifu 501-1193, Japan; The Research Institute for Time Studies, Yamaguchi University, 1677-1 Yoshida, Yamaguchi, Yamaguchi 753-8511, Japan; The Research Institute for Time Studies, Yamaguchi University, 1677-1 Yoshida, Yamaguchi, Yamaguchi 753-8511, Japan; Department of Biology, College of Liberal Arts and Sciences, Tokyo Medical and Dental University, Ichikawa, Chiba 272-0827, Japan; Department of Sport and Wellness, College of Sport and Wellness, Rikkyo University, Niiza, Saitama 352-8558, Japan; Department of Anatomy and Neurobiology, Kindai University, 377-2 Ohno-Higashi, Osaka-Sayama, Osaka 589-8511, Japan; RIKEN BioResource Research Center, 3-1-1 Koyadai, Tsukuba, Ibaraki 305-0074, Japan; Department of Cardiovascular Medicine, Saga University, 5-1-1 Nabeshima, Saga, Saga 849-8501, Japan; The Research Institute for Time Studies, Yamaguchi University, 1677-1 Yoshida, Yamaguchi, Yamaguchi 753-8511, Japan

**Keywords:** melatonin, aralkylamine *N*-acetyltransferase, cold adaptation, hibernation, Syrian hamster

## Abstract

A definitive understanding of intrinsic functions of endogenous melatonin may require genetic manipulation or modification of its synthesizing enzymes. Here, we established Syrian hamsters, a seasonal mammal, carrying loss of function of aralkylamine *N*-acetyltransferase (AANAT), a rate-limiting enzyme in melatonin biosynthesis. Mutants showed a normal circadian period but an accelerated entrainment to rescheduled light–dark cycles. We next focused on the role of melatonin in autumn/winter anticipation, given the strict increase in levels during short days. On exposure to cold after habituation to short days, all controls maintained normal core body temperature (*T*_b_), whereas many mutants showed a decrease in *T*_b_. Food shortage after this cold exposure induced hibernation in all controls and mutants; however, all mutants failed to continue a normal hibernation cycle, probably due to impaired *T*_b_ elevation during arousal from deep hibernation. These failures were accompanied by a decreased volume of lipid droplets in the interscapular brown adipose tissue (iBAT). Histological analyses of the pars tuberalis suggested a defect in photoperiodic responsiveness in mutants. Taken together, these findings demonstrate that defective photoperiodic responsiveness caused delayed remodeling of the iBAT in mutants and that sudden exposure to autumn/winter conditions caused severe defects in *T*_b_ homeostasis and interbout arousal, in both of which iBAT-mediated nonshivering thermogenesis plays a major role. AANAT-mediated melatonin biosynthesis appears to be indispensable to the survival of wild seasonal mammals in natural settings.

**Significance Statement**

To further define the role of the pineal hormone melatonin in seasonal adaptation, we established Syrian hamsters carrying loss of function of aralkylamine *N*-acetyltransferase, a rate-limiting enzyme in melatonin biosynthesis. Because the daily duration of melatonin secretion becomes longer as day length becomes shorter, we focused on autumn/winter adaptation and performed phenotypic analyses under cold and food shortage conditions. On exposure to cold after habituation to short days, all controls maintained normal core body temperature (*T*_b_), whereas many mutants showed a decrease in *T*_b_. Food shortage after this cold exposure induced hibernation in all controls and mutants; however, all mutants failed to continue a normal hibernation cycle. Melatonin therefore appears indispensable for survival of seasonal mammals in natural settings.

## Introduction

Aralkylamine *N*-acetyltransferase (AANAT) is a rate-limiting enzyme in melatonin biosynthesis (1–3). Specifically, melatonin is produced from serotonin (5-hydroxytryptamine) through the catalytic action of two key enzymes, AANAT and hydroxyindole-*O*-methyltransferase (HIOMT). Experimental manipulation of AANAT activity therefore enables elucidation of the in vivo function of melatonin. Numerous studies have shown that melatonin, an amine hormone which was previously called a wonder drug, is present not only in animals but also in plants and has a wide range of beneficial functions for organisms, including humans (4, 5). The major physiological functions of endogenous melatonin in mammals have been considered modulation of circadian rhythms and expression of seasonal adaptation, acting via highly expressed receptors in the suprachiasmatic nucleus (SCN) and pars tuberalis, respectively (6–11). These two functions are well consistent with its manner of secretion: melatonin is secreted from the pineal gland during dark periods under control of the circadian oscillator (12, 13). On the basis of this accumulating knowledge about the role of melatonin in mammalian circadian rhythms, exogenous melatonin and its derivatives are increasingly utilized as a supplement for sleep problems and jetlag (14–16).

In addition to its role in circadian rhythms and seasonal adaptation, pleiotropic functions of melatonin such as an antioxidant activity have been reported, mostly based on pharmacological experiments using exogenous melatonin, its derivatives, and receptor agonists and antagonists. Pharmacological approaches provide several experimental advantages in studying the role of melatonin: exogenous melatonin can be administered to any type of organism, without skilled techniques, and at any specific timepoint. Indeed, these approaches have greatly contributed to revealing potential intrinsic functions of melatonin and its medical benefits. However, as described above, endogenous melatonin is secreted strictly during dark periods, under control of the circadian oscillator and at very low plasma concentrations. Although these characteristics are important and nonnegligible for expression of the intrinsic physiological functions of endogenous melatonin, it is nearly impossible to reproduce them in pharmacological settings. Given that both the direction and strength of exogenous melatonin action depend on administration timing and dose (17, 18), interpreting whether the results obtained from pharmacological experiments truly reflect intrinsic aspects of endogenous melatonin is therefore difficult. On the contrary, the pineal gland is surgically resectable in several mammals, including laboratory rodents, and although the procedure is admittedly highly invasive, the results obtained using this approach more directly reflect functional aspects of endogenous melatonin. Against this, however, surgical removal of the whole organ will affect other physiological functions of the pineal gland separate to melatonin secretion. Taken together, notwithstanding numerous studies showing a wide range of potential functions of melatonin, these methodological limitations call into question whether endogenous melatonin really exerts all or even a part of these activities in vivo.

A definitive and robust understanding of the intrinsic functions of endogenous melatonin may therefore require an alternative and compensating experimental approach: specifically, genetic loss of function of key synthetic enzymes, such as AANAT and HIOMT. However, many laboratory mouse strains, including C57BL6, the major inbred strain used in genetic manipulation and modification studies, have lost the ability to synthesize and secrete melatonin during domestication (19, 20). To overcome this limitation, congenic mice were recently established by crossing melatonin-proficient and spontaneous melatonin-deficient mouse lines, and the use of these mice has allowed a more precise definition of the true functions of endogenous melatonin (21, 22). However, because laboratory mice do not show clear seasonal responses in physiology and behavior, they may not be appropriate for genetic investigation of the intrinsic function of endogenous melatonin in seasonal adaptation. To our knowledge, no studies have used genetic manipulation or modification in seasonal mammals, such as targeted gene disruption, to understand the mechanism of seasonal adaptation.

Here, to further define the intrinsic function of melatonin in mammalian seasonal adaptation using a genetic approach, we generated a seasonal mammal, *Mesocricetus auratus* (Syrian or golden hamster), lacking functional AANAT. Because it was technically difficult to extend traditional genetic methods to these hamsters, as suggested by the limited number of studies using genetically modified hamsters to date, we adopted and modified an in vivo oviductal electroporation method to establish genome-edited Syrian hamsters. Many seasonal mammals sense day length in order to anticipate a coming season, a process called photoperiodism (23, 24). Melatonin has been considered indispensable to this process; specifically, the pars tuberalis senses day length by receiving melatonin and controls seasonal adaptation via the hypothalamus–pituitary axis. The daily duration of melatonin secretion becomes longer as day length becomes shorter, and we therefore performed phenotypic analyses under autumn/winter conditions, in other words cold and food shortage. In particular, we focused on a seasonal metabolic strategy that is indispensable for survival during severe winter—hibernation—during which the interscapular brown adipose tissue (iBAT), the major tissue for nonshivering thermogenesis, plays a pivotal role in arousal from deep hibernation state (25–28).

## Results

### Generation of Syrian hamsters harboring an impaired AANAT and phenotypic analyses of growth and development in these hamsters

**Fig. 1.**
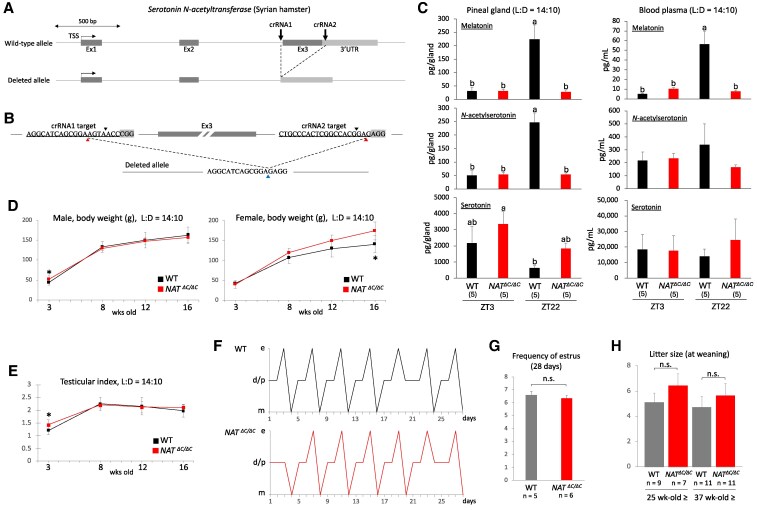
Generation of Syrian hamsters harboring an impaired AANAT and phenotypic analyses of growth and development in these hamsters. A) Schematic showing the genomic structure of the targeted wild-type and resulting deleted *AANAT* alleles in Syrian hamsters. Two arrows indicate the genomic sites targeted by two crRNAs. Exon 3 (Ex3) coding the C-terminal half of AANAT was deleted in the resulting allele. TSS, transcription start site; Ex, exon; UTR, untranslated region. B) The genomic sequence and position targeted by the two crRNAs designed to delete Ex3 in the *AANAT* allele. PAM (proto-spacer adjacent motif) sequences are shaded in gray. Double-strand break sites are indicated by black arrowheads. The resulting junction site and its putative original positions are indicated by blue and red arrowheads. C) Measurements of endogenous melatonin, *N*-acetylserotonin, and serotonin were performed with an LC–MS/MS method using the pineal gland and blood obtained from wild-type (WT) and homozygous (*AANAT^ΔC/ΔC^*) hamsters under a long-day condition. The number of hamsters used is shown in parentheses. Different alphabet letters represent significant differences (one-way ANOVA with Tukey's post hoc test, *P* < 0.05). LD, light and dark; ZT, zeitgeber time. D) Body weight of male and female WT and homozygous hamsters during growth (3 to 16 weeks old). Error bars indicate the mean ± SD. *P < 0.05. E) Length and width of the testis were measured during growth in live animals, and a testicular index was calculated. Error bars indicate the mean ± SD. *P < 0.05. F) Cytological assessment of estrous cycling performed by sequential collection of vaginal smears from WT and homozygous females for a period of 28 days. Representative results are shown as a line chart. The normal order of estrous stages is diestrus/proestrus (d/p), estrus (e), and metestrus (m). G) Frequency of estrus during 28 days was compared between WT and homozygous females. The number of hamsters used is shown below the graph. Error bars indicate the mean ± SD. n.s., not significant. H) Litter size of sexually matured mutant females was compared between WT and homozygous females at two different ages. Error bars indicate the mean ± SD. n.s., not significant.

To further define the role of melatonin in seasonal adaptation using a hitherto unused experimental approach, we aimed to generate Syrian hamsters lacking functional AANAT. Because we found it nearly impossible to perform in vitro manipulation of their oocytes and zygotes without death, we decided to apply an improved GONAD method (29–31), namely an in vivo oviductal electroporation method for in vivo genome editing of a target gene, to zygotes or two-cell embryos of Syrian hamsters. To determine an optimal electrical condition in which to successfully apply this method to these hamsters, we repeatedly performed oviductal electroporation with a variety of electric field intensities at the stage of poring pulse (Tables S1 and S2). As a result of PCR and sequencing screening of the F0 generation, we finally obtained an F0 hamster carrying an AANAT gene allele lacking the third exon coding the C-terminal half of AANAT (Figs. S1 and 1A). To prevent the potential risk that illegitimate translation may produce truncated but partially active forms of AANAT, we decided not to introduce an artificial error around the major translation start site but rather to delete the third exon containing residues directly binding to acetyl coenzyme A and a histidine residue essential for the catalytic activity. The resulting edited allele was well consistent with the target sites of two crRNAs we designed (Fig. 1B). Serotonin cannot be catalyzed to *N*-acetylserotonin in the pineal gland of homozygotes because the AANAT activity is lost due to the lack of its C-terminal half. To confirm this, we measured melatonin, *N*-acetylserotonin, and serotonin contents in the pineal gland of homozygotes obtained after backcrossing with wild-type hamsters for two generations using an liquid chromatography (LC)–mass spectrometry (MS)/MS method (Fig. 1C, left). As expected, while mutants showed a much lower melatonin content than controls at ZT22, around when its levels peaked in controls, mutants showed a similar low content to controls at ZT3, around when levels reached a trough in controls. Given that measurements of *N*-acetylserotonin showed similar results to those of melatonin and that serotonin likely accumulated in mutant pineal glands, it is certain that the decrease in melatonin content was attributable to the loss of AANAT activity. The decrease in melatonin levels at ZT22 was also detected in mutant blood plasma using the same LC–MS/MS method (Fig. 1C, right). The significant decrease in plasma melatonin concentration at ZT22 was reproduced by an ELISA method (Fig. S2). Unexpectedly, melatonin levels exceeded detection limits in both mutant blood plasma and pineal glands. In the temporal change of body weight during growth, while wild-type and mutant males showed no clear difference, mutant females became heavier than wild-type females as they grew (Fig. 1D). Testis index, an index of spermatogenesis, showed no obvious difference between wild-type and mutant males during growth (Fig. 1E), and estrous cycles showed no obvious difference in stage transition or estrus frequency between wild-type and mutant females (Fig. 1F and G). The litter size of sexually matured mutant females was larger than that of wild-type females albeit without statistical significance (Fig. 1H). Male and female mutants did not show any visible abnormality in the body.

### Effect of loss of function of AANAT on circadian rhythms in Syrian hamsters

**Fig. 2.**
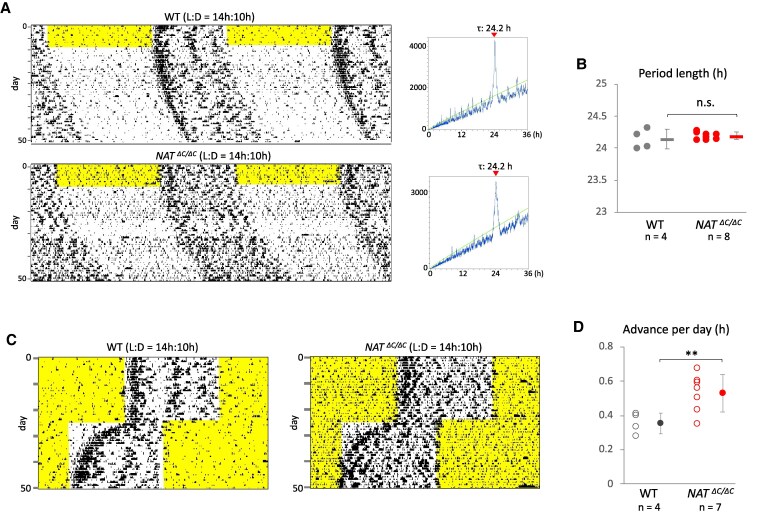
Effect of loss of function of AANAT on circadian rhythms in Syrian hamsters. A) Representative double-plotted free-running actograms of wild-type and homozygous mutant male hamsters. The light period is shaded in yellow. Corresponding periodograms drawn based on activity counts for the last 3 weeks are shown beside each actogram. A green line indicates *Q*_p_ (quasi-periodic) values at *P* = 0.05. The period length of the strongest *Q*_p_ peak is shown above the plot area. B) Free-running circadian period length of each wild-type and homozygous hamster was plotted. Dots and horizontal bars show individual data and their average, respectively. Error bars indicate the mean ± SD. n.s., not significant. C) Representative actograms showing circadian entrainment to rescheduled LD cycles. After habituation to regular 14:10-h LD cycles, wild-type and homozygous male hamsters were exposed to 6-h phase-advanced LD cycles. D) Average of circadian phase advance per day was calculated for a period from the onset day of rescheduled LD cycles to the day when circadian entrainment to the cycle was completed. Open and filled circles show individual data and their average, respectively. Error bars indicate the mean ± SD. **P < 0.01.

To examine the effect of impaired melatonin biosynthesis on the circadian rhythms of seasonal mammals, after isolation and habituation of male hamsters to regular 14-h light and 10-h dark (LD) cycles in a light-tight cabinet for more than 7 days, real-time monitoring of locomotor activity was performed under constant darkness using an infrared sensor (Fig. 2A, left). Circadian period length of free-running locomotor activity was calculated using a χ^2^ periodogram (Fig. 2A, right). No significant difference in circadian period length was detected between wild-type and mutant hamsters (wild-type, 24.14 ± 0.16 h; mutant, 24.19 ± 0.05 h) (Fig. 2B). To examine the speed of entrainment to rescheduled LD cycles, hamsters were exposed to 6-h advanced LD cycles (Fig. 2C). Previous reports on sheep and melatonin-proficient mice have demonstrated that the mammalian photoinducible phase exists within the range of the late dark phase, more specifically at around 12 h after the dark phase onset (32, 33). Under the present long-day condition before and after the 6-h phase advance, the photoinducible phase existed within the range of the light phase both before and after the advance. We can therefore exclude the potential risk that the change in photoperiod or difference in sensitivity to photoperiod between genotypes affected circadian phase shift. Mutants showed faster entrainment than wild-type hamsters in a statistically significant manner (wild-type, 0.36 ± 0.06 h/day; mutant, 0.53 ± 0.11 h/day) (Fig. 2D).

### Effect of loss of function of AANAT on adaptation to autumn-/winter-like conditions in Syrian hamsters

**Fig. 3.**
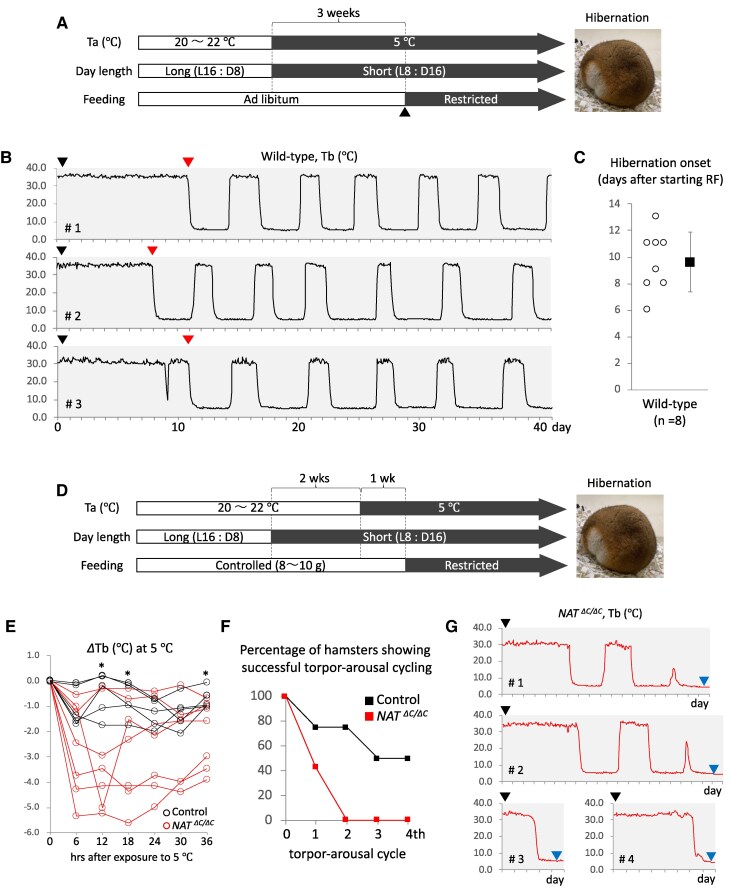
Effect of loss of function of AANAT on adaptation to autumn-/winter-like conditions in Syrian hamsters. A) Schematic representation of an experimental schedule simulating the transition from spring/summer (open) to autumn/winter (filled) conditions. Restricted feeding (gradual food shortage) was scheduled as follows: 10, 5, and 3 g of a regular laboratory chow were fed at the first, second, and following days, respectively. *T*_a_, ambient temperature. B) Representative data of core *T*_b_ from three wild-type hamsters for a period of 41 days. The first day is the start day of restricted feeding (black arrowhead). Red arrowheads show the day of hibernation onset. These *T*_b_ data clearly reflect repetitive deep hibernation and interbout arousal cycles (torpor–arousal cycles). C) Open circles represent the individual span from the first day of restricted feeding to the day of hibernation onset. Filled squares represent the average of these spans. Error bars indicate the mean ± SD. D) Schematic representation of a modified experimental schedule focusing on the role of photoperiodic anticipation in autumn/winter adaptation. Because hamsters with more than 140 g of body weight frequently failed hibernation regardless of genotype, daily food intake until the start of restricted feeding was adjusted with a range from around 8 to 10 g in a manner dependent on the body weight of individual animals. E) *T*_b_ relative to that at time 0 (Δ*T*_b_) in individual control and homozygous hamsters immediately after exposure to cold. *T*_b_ at time 0 was set to 0.0. Δ*T*_b_ was calculated at the 6-h interval by averaging four-timepoint *T*_b_ data that were measured at the 90-min interval. *P < 0.05 (controls versus homozygotes). F) Percentage of control and homozygous hamsters showing successful torpor–arousal cycling. When normal interbout arousal was observed after deep hibernation, the corresponding toper–arousal cycle was defined as “successful.” G) Representative *T*_b_ of homozygous hamsters which failed continuous hibernation. Black arrowheads indicate the start day of restricted feeding. Blue arrowheads indicate the timepoint when hamsters died. Hamsters were determined to be dead when no rise and fall of breathing were observed for two straight days. These hamsters were further confirmed to not show arousal after being exposed to room temperature.

The effect of impairment in melatonin biosynthesis on autumn/winter adaptation in seasonal mammals—namely adaptation to cold and food shortage—was examined using mutant hamsters. Before starting this assessment, we explored an environmental condition suitable for experimental induction of hibernation because Syrian hamsters are a facultative hibernator. Previous studies have shown that it takes around 3 months to experimentally induce hibernation under a cold and short-day condition (34, 35). To more appropriately simulate a natural autumn/winter condition, we further exposed hamsters to gradual food shortage 3 weeks after starting a cold and short-day condition (Fig. 3A). Almost all hamsters initiated hibernation around 10 days after exposure to the gradual food shortage (Fig. 3B and C), independent of sex and age. We also found that body weight at the start of the gradual food shortage strongly affected the success rate of normal and continuous hibernation cycling. Hamsters weighing around 115 to 135 g at the start of the gradual food shortage were therefore recruited for experimental induction of hibernation. Our preliminary trials indicated that a gradual food shortage long after the start of a cold exposure likely induced normal hibernation independent of genotype, probably via conventional cold adaptation processes independent of photoperiodic anticipation. To focus on the difference in photoperiodic responsiveness between genotypes, hamsters were therefore habituated to short days prior to cold exposure, shortly after which they were exposed to a gradual food shortage protocol (Fig. 3D). Data of core body temperature (*T*_b_) demonstrated that the cold exposure had little impact on *T*_b_ in any control but caused an obvious decrease in *T*_b_ in many mutants (Fig. 3E). A gradual food shortage 1 week after the start of a cold exposure induced hibernation in all controls and mutants. However, all mutants failed to continue hibernation cycles (Fig. 3F). The temporal change in *T*_b_ after hibernation onset suggests that several mutants failed in interbout arousal due to impaired thermogenesis (Fig. 3G).

### Effect of loss of function of AANAT on iBAT remodeling under an autumn-/winter-like condition and thyroid-stimulating hormone–mediated photoperiodic signaling

**Fig. 4.**
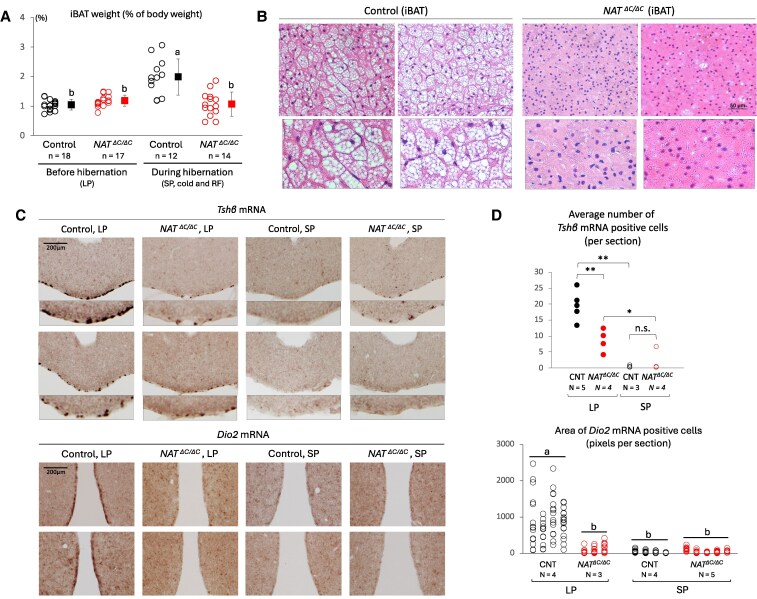
Effect of loss of function of AANAT on iBAT remodeling under an autumn-/winter-like condition and TSH-mediated photoperiodic signaling. A) The iBAT was harvested from control and *AANAT^ΔC/ΔC^* hamsters before and during hibernation. The wet weight of the iBAT was normalized to the body weight. Open circles and filled squares show individual data and their average, respectively. Error bars indicate the mean ± SD. Different alphabet letters represent significant differences (one-way ANOVA with Tukey's post hoc test, *P* < 0.05). B) Representative images of HE-stained paraffin sections of the iBAT from two independent control hamsters that had successful hibernation cycles and two independent homozygous hamsters that failed continuous hibernation. Vacuole-like structures are lipid droplets. The scale bar represents 50 um. The bottom boxed images show a magnified view (×2 magnification). C) Representative images of *Tshβ* probe-labeled sections of the pars tuberalis (top panels) and *Dio2* probe-labeled sections of the ependymal layer of the third ventricle (bottom panels), respectively, which were obtained from two independent control and homozygous hamsters. After all hamsters were habituated to a long photoperiod (LP, 14:10-h LD cycles), they were exposed to a short photoperiod (SP, 10:14-h LD cycles) for a period of 2 weeks. Serial coronal sections were processed using an in situ hybridization method. Digoxigenin was detected with alkaline phosphatase (brown signal). The lower images in the top panels show a magnified view of the basal region (×2 magnification). D) *Tshβ* mRNA-positive cells in the pars tuberalis were counted for each section, typically three to five sections in total per brain, in a single-blind and repeated manner (top graph). *Dio2* mRNA-positive area in the ependymal layer of the third ventricle was quantified for each section, typically in 13 to 15 sections in total per brain, using the ImageJ software (bottom graph). N represents the number of hamsters used in each experimental group. Multiple comparisons were performed using one-way ANOVA with Tukey's post hoc test. **P < 0.01; *P < 0.05; n.s., not significant (top graph). Different alphabet letters represent significant differences (one-way ANOVA with Tukey's post hoc test, *P* < 0.05) (bottom graph).

Because previous studies have shown that iBAT-mediated nonshivering thermogenesis plays a major role in *T*_b_ homeostasis under cold and interbout arousal from deep hibernation (25–28), we hypothesized that mutant-specific defects were caused by an insufficient remodeling of the iBAT. Supporting this hypothesis, during hibernation, the wet weight of the iBAT of control hamsters was significantly heavier than that of mutant hamsters (Fig. 4A). A similar result was obtained after exposure to the 2-week short-day condition without cold or food shortage (Fig. S3). Compared with control hamsters, hematoxylin and eosin (HE) images of iBAT tissue of mutant hamsters showed a smaller size of lipid droplets (Fig. 4B).

In mammalian photoperiodic signaling in the brain, a longer day length (shorter duration of melatonin secretion) increases expression of the thyroid-stimulating hormone β-subunit (TSHβ) in the pars tuberalis and resulting secretion of thyroid-stimulating hormone (TSH; heterodimer of α- and β-subunits) from the pars tuberalis, and the hypothalamus expresses type II iodothyronine deiodinase (Dio2) mainly in the ependymal layer of the third ventricle on receipt of the TSH signal. These pathways control the physiological and behavioral adaptations to spring/summer (36–39). Based on this theory, we examined expression levels of *Tshβ* mRNA in the pars tuberalis and *Dio2* mRNA in the ependymal layer using an in situ hybridization method (Fig. 4C). While the number of *Tshβ* mRNA-positive cells and the area of *Dio2* mRNA-positive cells were significantly larger in controls than that in mutants under a long-day condition, there was no significant difference between genotypes under a short-day condition (Fig. 4D).

## Discussion

### Targeted disruption of the *AANAT* gene in Syrian hamsters

A robust understanding of biological processes at the molecular level requires in vivo manipulation or modification of specific genes. However, to our knowledge, no studies have used these kinds of experimental approaches in seasonal mammals to understand the mechanism of seasonal adaptation. The major reason for this is the technical difficulty of extending traditional genetic methods to seasonal mammals. For example, only around 10 studies in total have applied these methods to Syrian hamsters (40–43), which are frequently used in research on mammalian seasonal adaptation, and none of these previous reports involved research to understand seasonal adaptation. Although a few previous studies have explained mechanistic aspects of mammalian seasonal adaptation using spontaneous mutant and genetically modified mice (44, 33), laboratory mice may not be a suitable animal model for seasonal adaptation because they do not show obvious seasonal changes in physiology and behavior. In the present study, we successfully established genome-edited Syrian hamsters carrying AANAT without the C-terminal half by modifying and applying an in vivo oviductal electroporation method, through many trials and errors. Further modification of the present protocol is required to raise the percentage of neonates that carry a successfully edited genome. All neonates carrying an impaired AANAT were kept under the long-day condition of 14:10-h LD because they grow and develop during long days in natural settings. External characteristics and reproductive functions did not show obvious abnormalities, other than a significant increase in body weight in mutant females. These results demonstrate that melatonin biosynthesis may not play a major role in the growth and development of Syrian hamsters. However, sexually mature mutant females likely showed a larger litter size than wild-type females, albeit without statistical significance, which is consistent with findings observed in melatonin-deficient congenic mice (22).

### Role of AANAT in circadian rhythms of Syrian hamsters

Mammalian melatonin receptors are highly expressed in the SCN of the hypothalamus, the central circadian clock, and administration of exogenous melatonin modulates circadian phase in physiology and behaviors such as locomotor activity (45–47). On the contrary, endogenous melatonin biosynthesis in the pineal gland is under the neuronal control of the SCN (48, 49); the pineal gland and SCN therefore regulate each other in a humoral and neuronal manner, respectively. This reciprocal regulation increases robustness in circadian rhythm generation. The two major melatonin receptors, MT1 and MT2, have been suggested to potentially have different physiological functions: namely, melatonin likely plays a role in circadian robustness via MT1 and in circadian adjustment via MT2 (50, 51). Unexpectedly, while congenic mice lacking functional AANAT and HIOMT showed delayed entrainment in response to advanced LD cycles in a previous study (22), Syrian hamsters carrying a mutant AANAT gene showed accelerated entrainment in the present study; accordingly, loss or decrease in endogenous melatonin likely leads to impaired circadian adjustment or impaired circadian robustness in the former or latter, respectively. Free-running periods of circadian locomotor activity showed no significant difference between wild-type and mutant animals in both mouse and hamster experiments. Although the reason is presently unclear, future detailed investigations of the difference in absolute expression levels and/or quantitative ratio of MT1 and MT2 in the SCN would explain phenotypic differences between these two mutant animals.

### Role of AANAT in autumn/winter adaptation of Syrian hamsters

Many seasonal organisms sense day length in order to anticipate a coming season, a biological process called photoperiodism (23, 24). In mammals, light input to the retina is transduced to the pineal gland via the SCN, where day length is converted to the duration of melatonin secretion. This duration is subsequently sensed by the pars tuberalis, in which melatonin receptors are highly expressed (52, 53). Shorter or longer duration of melatonin secretion increases or decreases expression of TSHβ and secretion of TSH from the pars tuberalis (36, 37). The hypothalamus—sensing a change in TSH signal intensity—controls physiology and behavior to adapt to spring/summer or autumn/winter by modulating the secretion of hormones from the pars distalis. On the contrary, although the responsible factor(s) tuberalin remains undefined, the pars tuberalis has been shown to directly modulate secretion of hormones from the pars distalis, independently of the hypothalamus (54, 55). A few pars tuberalis-derived substances have been identified as a potential tuberalin, for example, 2-arachidonoyl glycerol in Syrian hamsters and substance P and neurokinin A in sheep (54, 56). In summary, in the current model of mammalian photoperiodism, the pars tuberalis senses day length by receiving melatonin and enables seasonal adaptation by controlling the hypothalamus–pituitary axis through bidirectional signal transduction. Given these previous findings, we hypothesized that the loss of function of AANAT causes a decrease in responsiveness to short days, which leads to a delay in body remodeling to adapt autumn/winter. We therefore performed phenotypic analyses under autumn/winter conditions in Syrian hamsters lacking functional AANAT.

During autumn to winter, most wild animals are exposed to both cold and a shortage of food. To investigate the effect of a loss of function of AANAT on autumn/winter adaptation, we therefore exposed wild-type and mutant hamsters to not only cold but also food shortage. While previous reports reported that it took around 3 months for Syrian hamsters to initiate hibernation after exposure to cold and short days (34, 35), our present experiment demonstrates that food shortage strongly accelerates the onset of hibernation: namely, they initiated hibernation around 10 days after exposure to food shortage in addition to cold and short days. This demonstrates a significant and nonnegligible effect of food shortage on hibernation onset in this kind of hamsters, probably suggesting that feeding conditions should be taken into consideration to achieve a more natural induction of hibernation in at least some facultative hibernators. Food shortage long after exposure to cold induced a normal hibernation in most AANAT mutants in a manner similar to wild-type hamsters, probably because sufficient body remodeling had been achieved in time for hibernation onset through conventional cold adaptation processes independent of photoperiodic anticipation, suggesting that melatonin does not play a significant role in hibernation itself.

To focus on autumn/winter anticipation and remodeling initiation by photoperiod sensing, hamsters were therefore habituated to short days prior to cold exposure, shortly after which they were additionally exposed to food shortage, a potent trigger of hibernation as described above. Body weight or the amount of white adipose tissue is expected to strongly affect the impact of food shortage on cold adaptation, and this hypothesis is consistent with a previous report indicating that Syrian hamsters weighing over 140 g likely do not initiate hibernation under cold and short-day conditions (57). We therefore selected and recruited hamsters weighing around 115 to 135 g on the day of initiation of a food shortage protocol for the experimental induction of hibernation. Under exposure to cold after habituation to short days, all control hamsters maintained nearly constant and normal core *T*_b_. In contrast, many mutant hamsters showed an obvious decrease in *T*_b_. This strongly demonstrates that normal melatonin biosynthesis is necessary for the initiation of body remodeling for cold adaptation by photoperiodic anticipation of coming autumn/winter. We hypothesized that impaired body remodeling related to this mutant-specific phenotypic defect was an insufficient functional enhancement of BAT, the major tissue responsible for nonshivering thermogenesis (25, 26). Food shortage 1 week after the start of cold exposure induced the initiation of hibernation in all control and mutant hamsters. However, while half of the control animals continued normal hibernation cycles, all mutant animals failed. Importantly, the temporal change in *T*_b_ of mutant hamsters suggests that the failure was caused by impaired arousal from deep hibernation. Given reports that BAT-mediated thermogenesis plays a major and indispensable role in arousal from deep hibernation (27, 28), we again hypothesized that BAT remodeling was late for hibernation onset in animals with hibernation failure due to a defective photoperiodic response to short days.

To explore and explain the mechanism underlying impaired cold adaptation in mutant hamsters, we performed several histological analyses. In support of our hypothesis described above, the wet weight of the iBAT in mutant hamsters was indeed significantly smaller than that in control hamsters after exposure to a short photoperiod and during hibernation, and histological images further revealed that mutant hamsters showed a smaller lipid droplet size in the iBAT than control hamsters. These phenotypes of mutant hamsters are consistent with previous studies showing that administration of exogenous melatonin increases the volume of the iBAT (58). These histological findings strengthen our story that defective responsiveness to short days due to impaired melatonin biosynthesis caused delayed remodeling of the iBAT, and sudden exposure of mutant hamsters to cold and food shortage therefore caused severe problems in *T*_b_ homeostasis and interbout arousal, in both of which iBAT-mediated nonshivering thermogenesis plays a major role. Furthermore, to confirm this defective responsiveness to day length in mutant hamsters, we histologically detected expression levels of *Tshβ* mRNA in the pars tuberalis and *Dio2* mRNA in the ependymal layer of the third ventricle. Although previous studies suggested a pivotal role of endogenous melatonin in the mammalian photoperiodic retrograde pathway by comparing melatonin-proficient and melatonin-deficient mouse lines (44), the different genetic background of these lines limits interpretation. Our present histological data using mutant hamsters reproduced the previous results and therefore successfully compensate for this limitation. Given that a previous study using exogenous melatonin indicated that melatonin-induced suppression of *Tshβ* and *Dio2* expression enables photoperiodic responses to short days (59, 60), we expected that impaired endogenous melatonin biosynthesis would lead to up-regulation of *Tshβ* and *Dio2* expression under short-day conditions. However, mutant hamsters showed a significant difference in expression of *Tshβ* and *Dio2* mRNA compared with wild-type hamsters under not short-day but long-day conditions. These results well confirm that mammalian photoperiodic responses require melatonin biosynthesis, but also suggest that investigation of TSH signaling may be insufficient to directly evaluate the role of melatonin biosynthesis in photoperiodic anticipation of coming autumn/winter. Instead of TSH signaling, which was originally identified as a long-day pathway, well-defined molecular markers expressed in response to short days in the pars tuberalis are required, albeit that these have yet to be identified.

## Conclusion and limitations

In the present study, we present genetic evidence indicating that AANAT-mediated melatonin biosynthesis in seasonal mammals plays a significant role in photoperiodic anticipation of coming autumn/winter and timely initiation of body remodeling for adaptation to cold and food shortage. In natural settings, this biosynthesis would therefore be a vital biological process for wild seasonal mammals, given that their survival during severe winters absolutely requires the accurate anticipation of coming autumn/winter through sensitive sensing of photoperiodic changes for the initiation and completion of body remodeling without delay before exposure to severe cold and food shortage. However, note that our present results may not explain an indispensable role of melatonin biosynthesis in seasonal mammals having a circannual clock, because these animals can anticipate and adapt to coming seasons even without photoperiodic information (61–63). The present study also demonstrates that our in vivo oviductal electroporation method for manipulation or modification of specific genes in Syrian hamsters is powerful in seasonal biology and can therefore be used in future studies to reveal the true contribution of other known components related to seasonal adaptation. Nevertheless, a limitation of the present study should be mentioned: unexpectedly and surprisingly, we found that plasma concentrations of melatonin reached above background levels in Syrian hamsters carrying mutant AANAT, which may have hindered phenotypic comparison with wild-type hamsters. Consistently, minor concentrations of melatonin were also detected in congenic melatonin-proficient mice carrying mutated AANAT, but not in those with both mutated AANAT and HIOMT (21). Because low levels of melatonin were detected in not only blood plasma but also the pineal gland, all or at least some melatonin was highly likely biosynthesized in mutant hamsters. While a feeding-induced increase in plasma melatonin levels requires continuous feeding of a high-melatonin diet (64, 65), mutants were fed with a standard diet, as were controls. Our hypotheses for AANAT-independent minor melatonin biosynthesis in mutants are as follows. First, given that serotonin likely accumulated abnormally in mutant pineal glands, other *N*-acetyltransferases might slightly compensate for the loss of AANAT activity, albeit that serotonin is not a natural substrate for these enzymes. The *N*-acetyltransferases belonging to the GCN5-related *N*-acetyltransferase superfamily possess a conserved Ac-CoA binding domain and share a common mechanism of acetyl transfer (66). Second, *N*-acetylserotonin could be taken up into the pineal gland and be used for HIOMT-mediated melatonin biosynthesis. Mutants showed a significant and drastic decrease in *N*-acetylserotonin levels in the pineal gland, but not in blood plasma, strongly indicating that *N*-acetylserotonin was biosynthesized outside the pineal gland. As an additional hypothesis, deletion of the C-terminal half of AANAT may be insufficient for complete loss of its catalytic activity. However, this possibility is markedly low given its crystal structure, as previously reported (67, 68): specifically, deletion of the third exon results in loss of three α helices (α3 to α5) and four β sheets (β5 to β8) containing residues which directly bind to acetyl coenzyme A as well as a histidine residue essential for the catalytic activity of AANAT.

## Methods

### Animals

Outbred Syrian hamsters were commercially purchased from SLC Japan. The hamsters were housed singly in an animal facility with a regular 14:10-h LD cycle. The room temperature was maintained at around 21 °C, and food and water were supplied ad libitum. For breeding, the stage of the estrous cycle in female hamsters was sequentially monitored with a vaginal smear test, and estrous females and males were housed together for mating.

Throughout the entire experimental period, except for a period during and after hibernation induction, hamsters were fed ad libitum with MF (∼360 kcal per 100 g, Oriental Yeast), a standard pellet diet, whose melatonin content is not reported.

After birth, wild Syrian hamsters grow during a period of long days, namely spring and summer. Under laboratory conditions, neonates are therefore kept under a long-day condition to avoid the risk of being neglected or abnormal growth under a short-day condition. During their growth, we did not perform any phenotypic analyses under short-day conditions. There is a report indicating that young Syrian hamsters show no clear response to photoperiod (69).

All protocols for animal experiments were approved by the Animal Research Committee of Yamaguchi University. Animal studies were performed in compliance with the Yamaguchi University Animal Care and Use guidelines.

### CRISPR–Cas9 electroporation solution

In accordance with a previous study (70), to perform targeted disruption of the *AANAT* gene by genome editing, synthetic crRNA and tracrRNA were purchased as Alt-R CRISPR guide RNAs together with Alt-R S.p. Cas9 Nuclease 3NLS (Integrated DNA Technologies). The sequences of crRNAs designed in this study are listed in Table S1. To reconstitute each dried nucleic acid, crRNA and tracrRNA were suspended in RNase-free Duplex Buffer to a concentration of 200 μM. To prepare a well-structured RNA complex, equal volumes of crRNA and tracrRNA were mixed and denatured at 94 °C for 2 min and then placed at room temperature for 10 min. The appropriately structured crRNA and tracrRNA complex was further mixed with Cas9 protein so that the final concentration was 50 μM of crRNA/tracrRNA and 1 mg/mL of Cas9 protein. For visualization, fast green (Nacalai Tesque) was added to the mixture at a final concentration of 0.02%. The final mixture was diluted with Opti-MEM (Thermo Fisher Scientific) to adjust the volume to around 2 μL per oviduct and then used for oviductal electroporation, as follows.

### Oviductal electroporation

To introduce a loss-of-function mutation into the *AANAT* gene in Syrian hamsters, an in vivo genome-editing method called *i*-GONAD (improved-Genome editing via Oviductal Nucleic Acids Delivery) was performed according to a previously reported protocol (29–31, 70), with minor modifications. Briefly, female estrous hamsters were mated with male hamsters. Pregnancy onset was given a convenient definition of 0:00 on day 0. Female hamsters showing the presence of sperm in vaginal smears were anesthetized with isoflurane gas to perform oviductal electroporation under a dissecting microscope at 12:00 (day 0.5), the timepoint approximately corresponding to the late one-cell stage. After making an incision at the dorsal skin, the ovary–oviduct–uterus complex was carefully exposed outside the body. Around 2 μL of electroporation solution prepared above was injected into the hamster oviduct lumen from upstream of the ampulla using an ultrafine glass micropipette. Immediately after injection, the oviduct was completely covered with a piece of Kimwipe (Jujo-Kimberly) well soaked in phosphate-buffered saline and then grasped with tweezer-type electrodes. Oviductal electroporation was performed using the square-wave pulse generator CUY21EDIT II (BEX). We basically followed the electroporation parameters originally reported in mice (29, 30), but with a range of electric fields at the stage of poring pulse to survey optimal conditions for Syrian hamsters. After electroporation, the ovary–oviduct–uterus complex was carefully returned to its original position, and the dorsal incision was sutured and treated with an antibiotic. The hamsters were warmed until recovery from anesthesia. To identify neonates harboring a successfully edited genome, genotyping-based screening was conducted using genomic DNA from ear auricle tissue of F0 hamsters: genomic DNA was roughly extracted using a hot alkaline method and subjected to PCR, and the products were digested with restriction enzymes to distinguish whether each product originated from a wild-type or mutant allele. The PCR primers used are listed in Table S1. After definitive confirmation of the targeted allele by sequencing, selected F0 hamsters were back-crossed with wild-type hamsters two times and heterozygotes were further crossed with each other to obtain homozygotes.

### Melatonin measurement using LC and MS

Syrian hamsters were kept under a long-day condition (14:10-h LD). Under dim red light (< 30 lux), Syrian hamsters were euthanized after being anesthetized with isoflurane, and the eyes were immediately enucleated. The pineal gland and blood plasma were collected at ZT3 and ZT22, a timepoint when melatonin concentrations peak in this kind of hamster (71). These samples were stored at −80 °C until subsequent analysis. Melatonin and *N*-acetylserotonin, an immediate precursor of melatonin, were measured in the pineal gland and blood plasma in accordance with an LC–MS/MS protocol (72). Briefly, pretreated samples were applied to the liquid chromatography system AC30AD (Shimadzu) equipped with a C18 2.0 × 150-mm, 3-μm Kinetex column (Tosoh). A solution of 10 μM ammonium acetate in 0.05% (v/v) acetic acid with varying concentrations was used as a mobile phase. A linear gradient was performed with 5 to 50% MeOH for 20 min or more and then with 100% MeOH for 10 min. Flow rate was set to 0.3 mL/min, and the column oven and auto sampler were kept at 25 and 4 °C, respectively. Melatonin and *N*-acetylserotonin were identified with a triple quadrupole mass spectrometer LCMS-8050 (Shimadzu), and quantitative analysis was performed by multiple reaction monitoring of the transition of parent ions to product ions.

### Melatonin measurement using an ELISA method

Blood was obtained by cardiac puncture and collected in a heparinized tube. Plasma was collected after centrifugation, flash-frozen with liquid nitrogen, and stored at −80 °C until use. Plasma concentrations of endogenous melatonin were measured using an ELISA method in accordance with manufacturer's instructions (Melatonin ELISA, IBL International).

### Reproductive function

The maximum length and width of the testis of male hamsters were measured during growth without killing using calipers. An index of testis size was calculated as follows: length × width ÷ body weight. This index was previously reported to be highly correlated with the total weight of both testes relative to body weight (73). Testis weight itself is known to be an excellent index of spermatogenesis (74).

To investigate estrous cycling, cytological assessment was performed by sequential collection of vaginal smear samples. Specifically, vaginal specimens were collected from female hamsters by careful and gentle wiping of the vaginal opening with a cotton swab soaked in saline and then transferred to a dry glass slide. The smear samples on slides were air-dried and then stained with 1:25 diluted Giemsa stain solution (Wako). To define estrous cycle stage, the smear samples were microscopically and cytologically assessed based on the predominance of leukocytes, cornified epithelial cells, and nucleated epithelial cells.

### Locomotor activity

Male hamsters aged 16 to 24 weeks were kept singly in a standard cage placed in a compartment equipped with an infrared sensor to detect locomotor activity under free-moving conditions. Activity counts were recorded in real time using the software Clock Lab (Actimetrics). Hamsters were allowed ad libitum access to food and water, which were exchanged with fresh food and water every 2 weeks. After habituation to the environment inside a compartment, hamsters were entrained to a 14:10-h LD cycle for around 2 weeks and then exposed to a 6-h phase-advanced LD cycle. To calculate the average of circadian phase advance (h) per day, the average of circadian period length of locomotor activity was calculated for the period from the onset day of the rescheduled LD cycle to the day when circadian entrainment to the cycle was completed using the software MATLAB. After completion of entrainment to the rescheduled cycle, a constant darkness condition was started and continued for more than 6 weeks. The free-running circadian period length was calculated for each animal by a χ^2^ method based on activity counts from the last 3 weeks using the software ActogramJ (75).

### Core *T*_b_

Syrian hamsters were anesthetized with isoflurane gas and an iButton (Thermochron Type-G, KN Laboratories) was surgically implanted in the abdominal cavity for core *T*_b_ recording. We programmed the iButtons to record the temperature every 90 min and waterproofed them with clear plastic tool dip (Plasti Dip International) before implantation. Data were read out using the software ThermoManager (KN Laboratories).

### Cold and food shortage

To exclude the effect of the difference in body weight on the success rate of hibernation, healthy-looking hamsters of around 115 to 135 g were recruited for experimental induction of hibernation. To simulate a natural autumn/winter condition, hamsters were experimentally exposed to gradual food shortage in addition to cold (5 °C) and short-day (8:16-h LD) conditions. During the period of gradual food shortage, hamsters were fed with 10, 5, and 3 g of sunflower seeds on the first, second, and following days, respectively. To determine that hamsters were alive during deep hibernation, the rise and fall of their breathing were confirmed visually or through video recording for a period of around 5 min. Hamsters were considered to be dead when no rise and fall were observed for two straight days and the iBAT was immediately harvested for the histological analyses. Definitive confirmation of death requires successive observation of hamsters for 2 days, due to the difficulty in distinguishing between the dead and deep hibernation states. We confirmed using euthanized hamsters that this period, 48 h after death under cold conditions, had little effect on either wet weight or histological imaging of the iBAT.

### Histological analysis

Hamsters were humanely killed using isoflurane, and iBAT specimens were collected and used to evaluate the volume of lipid droplets by HE staining. In accordance with a conventional protocol, iBAT specimens were fixed in paraformaldehyde, dehydrated with increasing concentrations of ethanol, cleared with xylene, and then embedded with paraffin. Paraffin blocks were sectioned using a rotary microtome. Paraffin sections were placed on a slide glass and deparaffinized with xylene and then sequentially with decreasing concentrations of ethanol. HE staining was performed according to a standard method. After staining, the sections were dehydrated and enclosed under a cover glass. A magnified view of sections was photographed using a conventional optical microscope, and the digital images were stored.

Coronal brain sections containing the entire rostral to caudal extent of the pars tuberalis and the ependymal layer of the third ventricle were cut at 30 um using a cryostat and processed in accordance with a free floating in situ hybridization method (76). In situ hybridization was performed on these sections using digoxigenin-labeled *Tshβ* and *Dio2* cRNA probes corresponding to *Tshβ* cDNA (+22 to +474) and *Dio2* cDNA (+46 to +472) (+1 is the translation start site), which were synthesized using a standard protocol for cRNA synthesis in accordance with the manufacturer's instructions (Roche Diagnostics Japan). The sequences of PCR primer sets to amplify cDNA templates for probes are listed in Table S3. Digoxigenin was detected with anti-digoxigenin antibody conjugated with alkaline phosphatase. As expected, no significant signals were detected using sense probes or following pretreatment of the sections with an RNase. *Tshβ* mRNA-positive cells were counted in each section, typically three to five sections in total per brain, in a single-blind manner. Because it was difficult to count *Dio2* mRNA-positive ependymal cells due to their vague cell outline, the total positive area was quantified using the ImageJ software in each section, involving typically a total of 13 to 15 sections per brain.

### Quantification and statistical analysis

In general, numeric data are represented as the mean ± SD or SE. The specific statistical tests used, *P*-value level definitions, and additional details are described in each figure legend.

## Supplementary Material

pgaf159_Supplementary_Data

## Data Availability

The data supporting the findings of this article are available within the article and/or its Supplementary Materials.
