# Supplementary material for: Targeted disruption of the aralkylamine N-acetyltransferase gene in a seasonal mammal, Mesocricetus auratus
Source: PNAS Nexus. 2025 May 20;4(6):pgaf159. doi: 10.1093/pnasnexus/pgaf159 (PMC12130684; doi:10.1093/pnasnexus/pgaf159)
Supplement: pgaf159_Supplementary_Data [file pgaf159_supplementary_data.pdf]

Genomic sequence targeted by *hamAANAT* crRNA1
5’- AGGCATCAGCGGAAGTAACCCGG -3’<br>(93531772 to 93531794, positive)
Genomic sequence targeted by *hamAANAT* crRNA2
5’- CTGCCCACTCGGCCACGGAGAGG -3’<br>(93532132 to 93532154, positive)

Forward primer for F0 screening
5’- TGCTGCTTTAGCGTCTCGAA -3’<br>(93531616 to 93531635, positive)
Reverse primer for F0 screening
5’- GGTCCTTTGGGGACAGATGG -3’<br>(93532327 to 93532346, negative)

Genotyping forward primer for WT allele
5’- AGGCATCAGCGGAAGTAACC -3’<br>(93531772 to 93531791, positive)
Genotyping reverse primer for WT allele
5’- TGCTGCTCCCCACCCTCTCCGT -3’<br>(93532146 to 93532167, negative)

Table S1

Nucleotide sequences of *AANAT* genomic regions targeted by crRNAs, F0 screening and genotyping primers

Although the primer set used for F0 screening (middle table) was able to amplify both the wild-type and edited *AANAT* alleles, the former were poorly amplified in comparison with the latter (see Figure S1). An additional primer set specific for the wild-type allele was therefore used for the subsequent generations (bottom table). Numbers in parentheses indicate the genomic position on the reference assembly BCM_Maur_2.0. “positive” and “negative” indicate the genomic direction or strand.

Poring pulse	No. of hamsters treated	No. of newborns obtained	No. of F0 hamsters with a mutated allele
100 mA	5	27	0
150 mA	3	8	0
180 mA	2	3	0
190 mA	3	9	0
50 V	14	31	1 (3.2%)

…

Table S2

Results from oviductal electroporation with a variety of electric conditions at the stage of poring pulse

Forward primer for the *Tshβ* probe template
5’- TCCGTGCTTTTTGCTCTTGC -3’<br>(53781955 to 53781974, positive)
Reverse primer for the *Tshβ* probe template
5’- TAGTTCTATTCCAGGTAAACACAT -3’<br>(53782808 to 53782831, negative)

Forward primer for the *Dio2* probe template
5’- CCAGTCTTTTTCTCCAACTG -3’<br>(27439656 to 27439675, positive)
Reverse primer for the *Dio2* probe template
5’- TGTATACCAACAGGAAGTCG -3’<br>(27447822 to 27447841, negative)

Table S3

Nucleotide sequences of PCR primer sets to amplify cDNA templates for *in situ* hybridization probes

Numbers in parentheses indicate the genomic position on the reference assembly BCM_Maur_2.0. “positive” and “negative” indicate the genomic direction or strand.

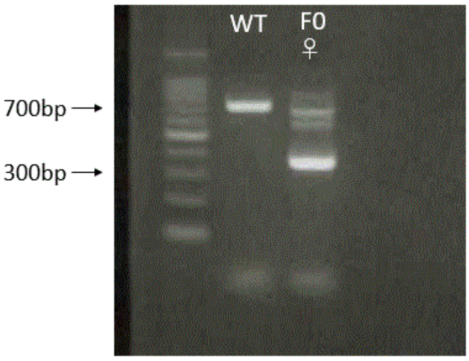

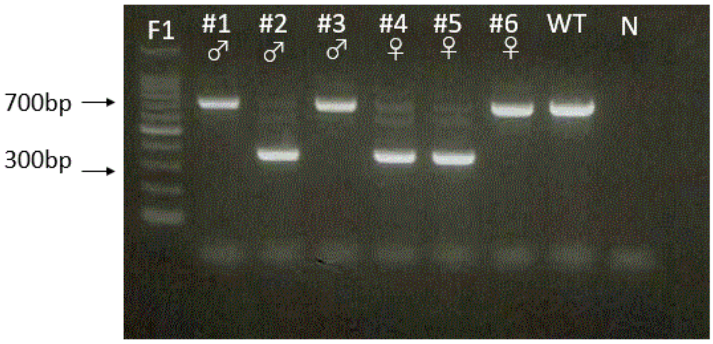

Figure S1

Screening of the F0 generation

(Top) F0 newborns were subjected to PCR-based screening. The gel image indicates the F0 hamster carrying an AANAT gene allele lacking the third exon, as shown in Table S2. A 100 ladder marker was loaded on the left-most lane. (Bottom) The number above each lane represents each F1 newborn. The PCR products located above the marker bands 700 bp and 300 bp were derived from the wild-type and genome-edited alleles, respectively. WT, wild type hamster; N, negative control sample for PCR.

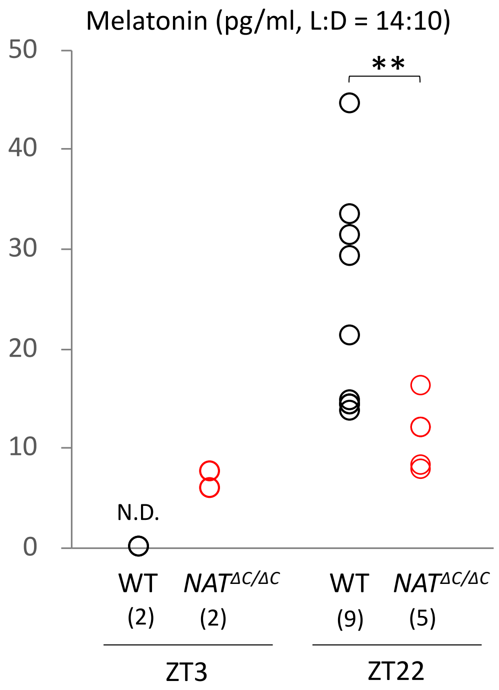

Figure S2

Measurement of plasma melatonin by ELISA

Measurements of plasma concentrations of endogenous melatonin were performed with an ELISA method using blood obtained from wild-type (WT) and homozygous (*AANAT*$^{\Delta C/\Delta C}$) hamsters by cardiac puncture. The number of hamsters used is shown in parentheses. **P < 0.01 (unpaired t test, WT vs. *AANAT*$^{\Delta C/\Delta C}$). LD, light and dark; N.D., not detected; ZT, zeitgeber time.

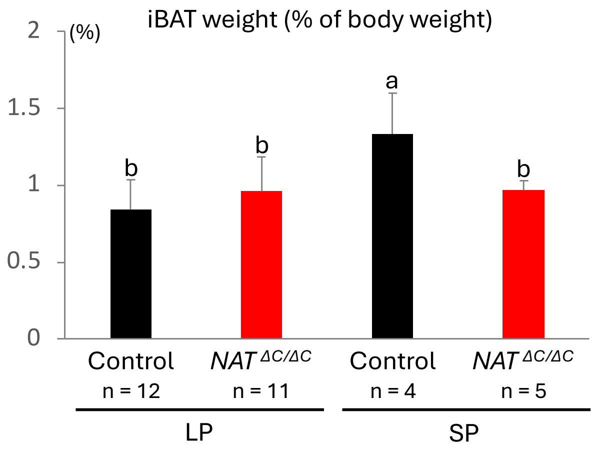

Figure S3

iBAT weight under long- and short-day conditions

iBAT was harvested from control and *AANAT*$^{\Delta C/\Delta C}$ hamsters under long (LP) and short photoperiod (SP) conditions. The wet weight of the iBAT was normalized to the body weight. Error bars indicate the mean ± SD. Different alphabet letters represent significant differences (one-way ANOVA with post hoc Tukey's test, $p < 0.05$).
